# Artificial intelligence-enabled 8-lead ECG detection of atrial septal defect among adults: a novel diagnostic tool

**DOI:** 10.3389/fcvm.2023.1279324

**Published:** 2023-11-13

**Authors:** Qiushi Luo, Hongling Zhu, Jiabing Zhu, Yi Li, Yang Yu, Lei Lei, Fan Lin, Minghe Zhou, Longyan Cui, Tao Zhu, Xuefei Li, Huakun Zuo, Xiaoyun Yang

**Affiliations:** ^1^ Division of Cardiology, Department of Internal Medicine, Tongji Hospital, Tongji Medical College, Huazhong University of Science and Technology, Wuhan, China; ^2^ Wuhan Zoncare Bio-Medical Electronics Co., Ltd, Wuhan, China; ^3^ Division of Cardiology, the Central Hospital of Wuhan, Tongji Medical College, Huazhong University of Science and Technology, Wuhan, China; ^4^ School of Medicine and Health Management, Tongji Medical College, Huazhong University of Science and Technology, Wuhan, China; ^5^ Wuhan National High Magnetic Field Center, Huazhong University of Science and Technology, Wuhan, China

**Keywords:** atrial septal defect, artificial intelligence, electrocardiogram, convolutional neural network, diagnosis among adults, 8-lead ECG

## Abstract

**Background:**

Patients with atrial septal defect (ASD) exhibit distinctive electrocardiogram (ECG) patterns. However, ASD cannot be diagnosed solely based on these differences. Artificial intelligence (AI) has been widely used for specifically diagnosing cardiovascular diseases other than arrhythmia. Our study aimed to develop an artificial intelligence-enabled 8-lead ECG to detect ASD among adults.

**Method:**

In this study, our AI model was trained and validated using 526 ECGs from patients with ASD and 2,124 ECGs from a control group with a normal cardiac structure in our hospital. External testing was conducted at Wuhan Central Hospital, involving 50 ECGs from the ASD group and 46 ECGs from the normal group. The model was based on a convolutional neural network (CNN) with a residual network to classify 8-lead ECG data into either the ASD or normal group. We employed a 10-fold cross-validation approach.

**Results:**

Statistically significant differences (*p* < 0.05) were observed in the cited ECG features between the ASD and normal groups. Our AI model performed well in identifying ECGs in both the ASD group [accuracy of 0.97, precision of 0.90, recall of 0.97, specificity of 0.97, F1 score of 0.93, and area under the curve (AUC) of 0.99] and the normal group within the training and validation datasets from our hospital. Furthermore, these corresponding indices performed impressively in the external test data set with the accuracy of 0.82, precision of 0.90, recall of 0.74, specificity of 0.91, F1 score of 0.81 and the AUC of 0.87. And the series of experiments of subgroups to discuss specific clinic situations associated to this issue was remarkable as well.

**Conclusion:**

An ECG-based detection of ASD using an artificial intelligence algorithm can be achieved with high diagnostic performance, and it shows great clinical promise. Our research on AI-enabled 8-lead ECG detection of ASD in adults is expected to provide robust references for early detection of ASD, healthy pregnancies, and related decision-making. A lower number of leads is also more favorable for the application of portable devices, which it is expected that this technology will bring significant economic and societal benefits.

## 1. Introduction

Atrial septal defect (ASD) is a common congenital heart disease characterized by direct communication between the atrial chambers. While many young adults with ASD may not exhibit symptoms, the condition can lead to serious complications such as arrhythmias, right heart failure, thromboembolism, and pulmonary arterial hypertension (PAH). The early detection and treatment of ASD is crucial for improving patient outcomes and survival rates (1, 2).

ASD can be classified into several types, including secundum, primum, sinus venosus, and coronary sinus defects. The majority of ASD cases (about 80%) are secundum ASD, located in the region of the foramen ovale, while 15% are primum ASD located in the lower portion of the atrial septum. In this study, we will focus on the secundum and primum defects as they account for 95% cases of ASD (3).

The standard diagnostic methods for ASD are transthoracic echocardiography or transesophageal echocardiography (TTE), which require specialized medical expertise and can be costly and difficult to implement widely. Previous studies have shown that patients with ASD exhibit different ECG patterns compared to individuals with normal cardiac structure, with typical findings including atrial tachyarrhythmias, incomplete right bundle branch block, a tall P-wave indicative of right atrial enlargement, right ventricular hypertrophy, and a notched R wave in leads II, III, and AVF (4). However, ASD cannot be diagnosed solely based on these differences.

Artificial Intelligence (AI) is a general term that implies the use of a computer to model intelligent behavior with minimal human intervention (5). Deep learning allows computational models that are composed of multiple processing layers to learn representations of data with multiple levels of abstraction (6). Convolutional Neural Network (CNN) is the widely used deep learning framework. CNN is made of convolutions having learnable weights and biases similar to neurons (nerve cells) of the animal, which has been widely used in artificial intelligence-ECG (AI-ECG) for specifically diagnosing cardiovascular diseases other than arrhythmia. The residual network which is an improvement of the traditional CNN is developed to increase the layers of the network (7, 8). In this study, we aimed to develop an AI-ECG algorithm for detecting ASD among adults, which contained a CNN with a residual network. Comparing to the previous studies by Moris et al. (9) and Kai Liu et al. (10) and Kotaro et al. (11), the novelty of our study lay in the utilization of artificial intelligence-enabled 8-lead ECG signal classification (with a lower computational load compared to 12-lead) for screening congenital heart disease in adults with comprehensive demographic and electrocardiographic data. And we also did a series of experiments of subgroups to discuss specific clinic situations associated to this issue. Meanwhile, dual-center validation yielded excellent results and increased the robustness of the model.

## 2. Patients and methods

### 2.1. Data collection

Our dataset consisted of retrospective data from patients aged ≥18 years who had an ECG at the Cardiac Function Examination Center of Tongji Hospital and the Wuhan Central Hospital (Huazhong University of Science and Technology, Wuhan, China).

Our research included two datasets from two centers: Tongji Hospital, affiliated with Tongji Medical College, Huazhong University of Science and Technology (Wuhan Tongji Hospital); and Wuhan Central Hospital. The ECGs from the former were standard 10 s, 12-lead recordings at a sampling rate of 500 Hz, obtained using a GE-Marquette ECG machine (GE MAC5500, GE Healthcare, Milwaukee, WI, USA). The ECGs from the latter were standard 10 s, 12-lead recordings at a sampling rate of 500 Hz, obtained on a Philips–Amsterdam machine or a Nihon Kohden-Tokyo ECG machine. In order to ensure the training speed of the model, we selected an 8-lead (III, avR, avL and avF were deleted in the standard 12-lead raw) as the input to the model.

**Figure 1:**
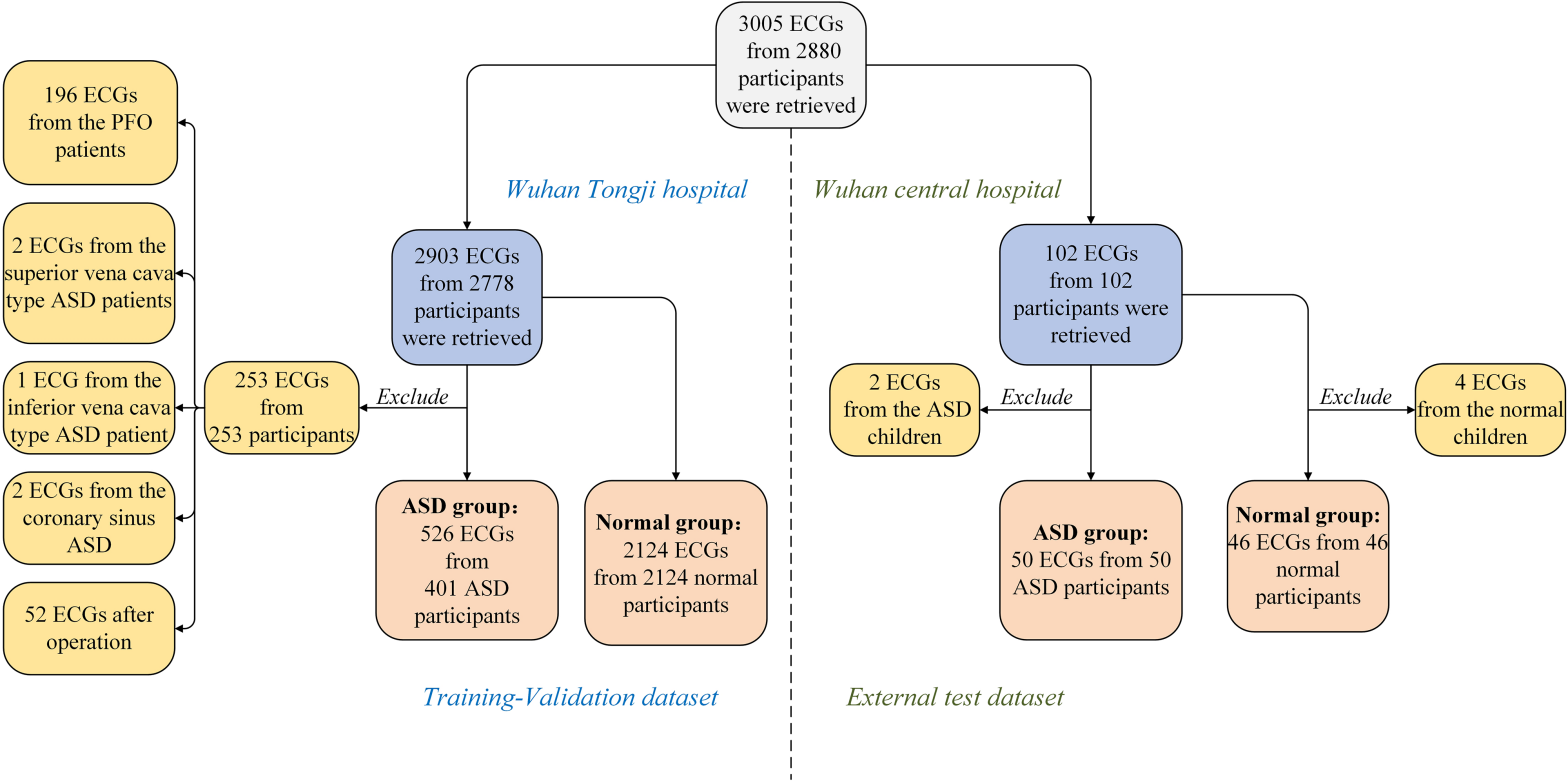
Study sample selection. Medical record database and ECGs database were obtained from Tongji Hospital, Tongji Medical College of Huazhong University of Science and Technology and Wuhan Central Hospital in 2012.01-2022.09. ASD, atrial septal defect; ECG, electrocardiogram.

As shown in Figure 1, from the two centers, there was a total of 2,880 participants, and 3,005 ECGs were retrieved. Data from Tongji Hospital, included 2,903 ECGs and 2,778 participants. After excluding cases of patent foramen ovale (PFO), less common types such as coronary sinus-type and sinus venosus-type ASD, and postoperative ECGs, our center included 401 participants with premium and secundum ASD and their corresponding 526 preoperative ECGs. The control group consisted of 2,124 participants and 2,124 ECGs, all of whom had normal cardiac structure confirmed via transthoracic echocardiogram. These two sets of data comprised the training and validation sets. Data from Wuhan Central Hospital included 102 ECGs and 102 participants. After excluding 2 ECGs with ASD and 4 ECGs with normal cardiac structure, a total of 50 participants with ASD and their corresponding 50 ECGs, as well as 46 participants with normal cardiac structure confirmed via transthoracic echocardiogram and their corresponding 46 ECGs, were included in the external test set, which consisted of a total of 96 ECGs.

We conducted a series of experiments to explore specific scenarios in our study. As shown in Table 1, the ASD group in the training-validation dataset included 121 normal ECGs, 224 ECGs with incomplete right bundle branch block (IRBBB), 86 ECGs with complete right bundle branch block (CRBBB), and 67 ECGs with right ventricular hypertrophy (RVH). After excluding the multi-label ECGs, our experimental group consisted of 191 ECGs with IRBBB only, 72 ECGs with CRBBB only, or 22 ECGs with RVH only. Meanwhile, we identified specific subgroups within the normal control group that displayed electrocardiogram patterns similar to those observed in the ASD group. These subgroups consisted of 1,514 normal ECGs, 30 ECGs with CRBBB, 53 ECGs with IRBBB, and 9 ECGs with RVH. We then proceeded to compare each of these subgroups from the normal control group with their corresponding electrocardiogram diagnostic subgroups in the ASD group.

**Table 1:** Differential analysis of the ECG features in the training-validation dataset and test dataset.

	Training-validation dataset			Test dataset		
	ASD group (*n* = 526)	Normal group (*n* = 2,124)	*P* value	ASD group (*n* = 50)	Normal group (*n* = 46)	*P* value
Mean age	41.9 ± 13.7	48.6 ± 15.3	**<0**.**001**	54.8 ± 16.3	44.3 ± 13.1	0.002
Female	307 (76.6)	1,173(55.2)	**<0**.**001**	37 (74.0)	31 (67.3)	0.508
Normal ECGs	121 (23.0)	1,514 (71.3)	**<0**.**001**	19 (38.0)	38 (82.6)	**<0**.**001**
Abnormal ECGs	405 (77.0)	610 (28.7)	**<0**.**001**	31 (62.0)	8 (17.4)	**<0**.**001**
Sinus rhythm	462 (87.8)	2,110 (99.3)	**<0**.**001**	43 (86.0)	46 (100.0)	0.013
Atrial rhythm	64 (12.2)	14 (0.7)	**<0**.**001**	7 (14.0)	0	0.013
1°AVB	30 (5.7)	11 (0.5)	**<0**.**001**	–	–	–
IRBBB	224 (42.6)	55 (2.6)	**<0**.**001**	9 (18.0)	1 (2.2)	0.017
CRBBB	86 (16.3)	34 (1.6)	**<0**.**001**	8 (16.0)	0	0.006
RVH	67 (12.7)	13 (0.6)	**<0**.**001**	1 (2.0)	0	>0.999
LVH	8 (1.5)	140 (6.6)	**<0**.**001**	0	1 (2.2)	0.479
ELLA	18 (3.4)	24 (1.1)	**<0**.**001**	2 (4.0)	0	0.496
ELRA	10 (1.9)	6 (0.3)	**<0**.**001**	1 (2.0)	0	>0.999
APB	17 (3.2)	35 (1.6)	0.019	2 (4.0)	0	0.496
VPB	24 (4.6)	35 (1.6)	**<0**.**001**	6 (12.0)	0	0.027

ASD, atrial septal defect; ECG, electrocardiogram; atrial rhythm, atrial fibrillation, atrial flutter, and atrial tachycardia; 1°AVB, first-degree atrioventricular block; IRBBB, incomplete right bundle branch block; CRBBB, complete right bundle branch block; RVH, right ventricular hypertrophy; LVH, left ventricular hypertrophy; ELLA, enlargement of the left atrium; ELRA, enlargement of the right atrium; APB, atrial premature beat; VPB, ventricular premature beat.

The bold values indicate statistically significant difference between the two groups.

**Figure 2:**
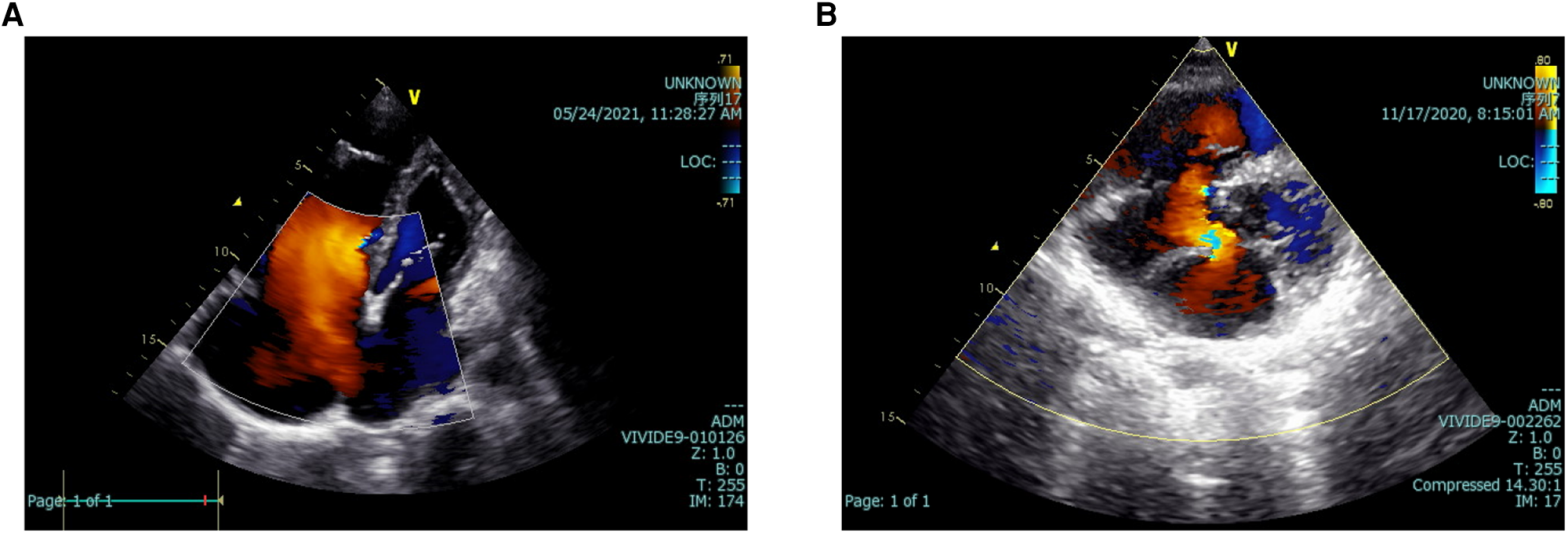
Echocardiograms of two kinds of ASD. The echocardiogram of secundum (**A**), and primum (**B**) ASD. Doppler ultrasound shows the direction and location of atrial shunting and the defect location. ASD, atrial septal defect.

**Figure 3:**
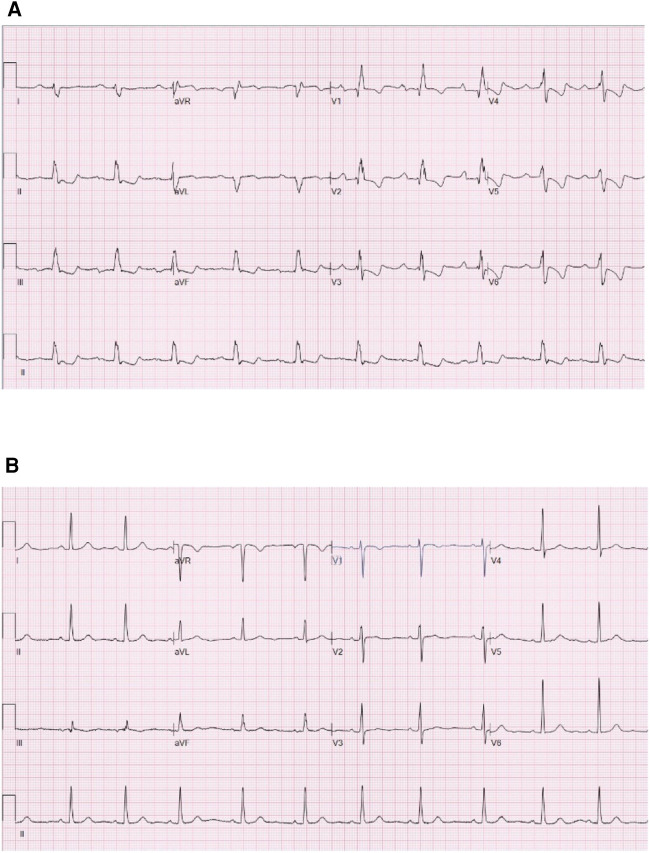
Electrocardiograms of the participants of the ASD group and the normal group. (**A**) Is the electrocardiogram of a participant of the ASD group. (**B**) Is the electrocardiogram of a participant of the normal group. ASD, atrial septal defect.

In addition, Figures 2, 3 depict imaging criteria for the basic materials. Meanwhile, Figure 2 shows the echocardiogram of secundum (Figure 2A), and primum (Figure 2B) ASD, in which doppler ultrasound shows the direction and location of atrial shunting and the defect location. The defect of the primum ASD is closer to the ventricle.

Meanwhile, Figure 3 shows the electrocardiograms of the participants of the ASD group (Figure 3A) and the normal group (Figure 3B). Figure 3A shows first-degree atrioventricular block, complete right bundle branch block, right ventricular hypertrophy and rightward deviation of the electrical axis. Meanwhile, Figure 3B displays a normal ECG.

### 2.2. Overview of the artificial intelligence model

#### 2.2.1. Distribution of the training–validation set and test set

After the AI algorithm from the training–validation set had been developed, this study included a total of 2,650 ECGs from Tongji Hospital, comprising 526 ECGs from 401 patients with ASD and 2,124 ECGs from the 2,124 subjects in the normal group. To create a combined training–validation set, we used ten-fold cross-validation, where each fold consisted of 1/10 of the data as the validation set and the remaining 9/10 as the training set. This approach generated independent algorithm results for each fold, and the scores were then summed to obtain the final score for AI-ECG performance. After that, the external test, which consisted of 96 subjects and 96 ECGs, was used to evaluate the performance of the model (12).

#### 2.2.2. CNN with residual networks

**Figure 4:**
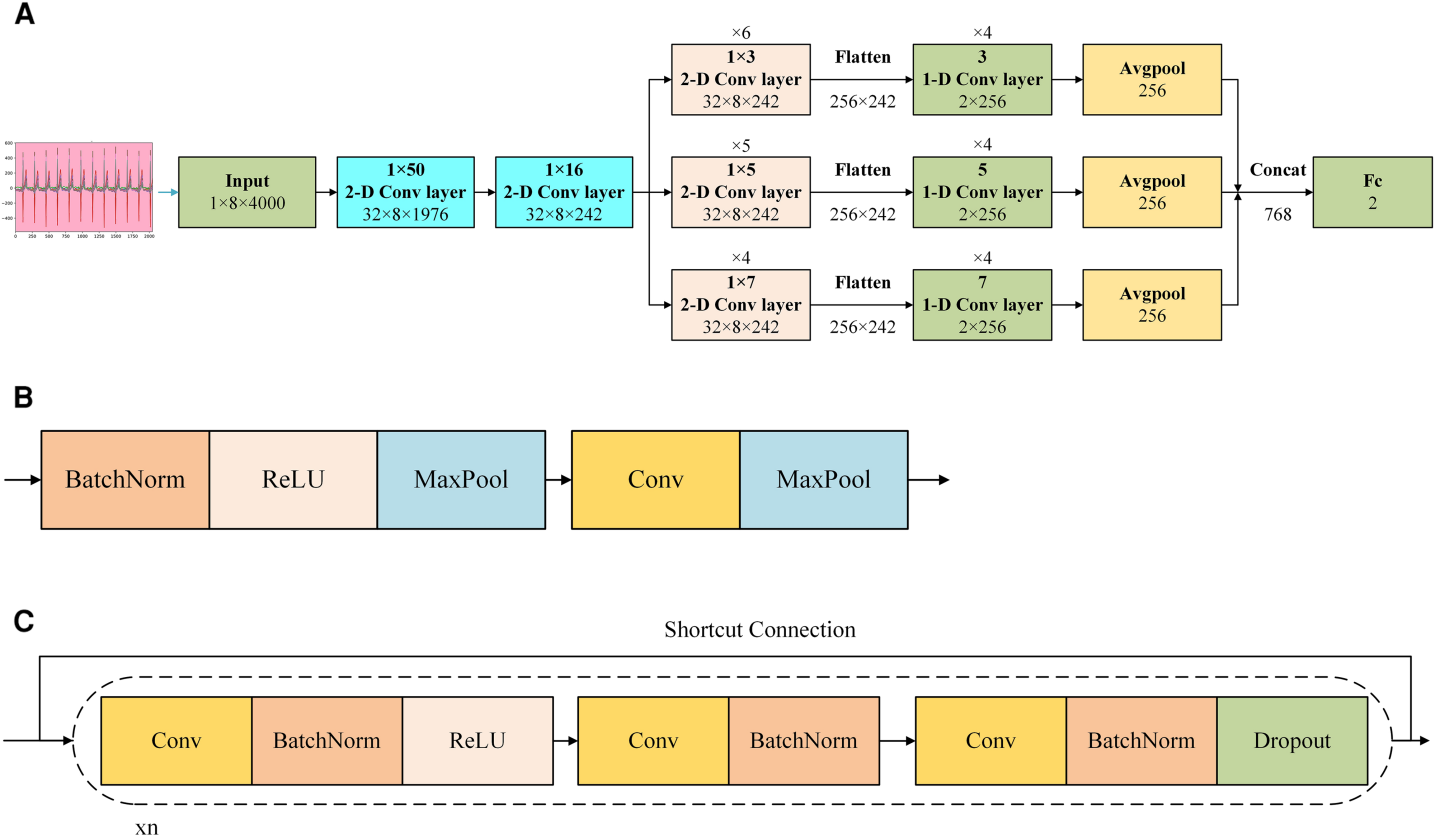
Demonstration of AI model of the CNN with residual networks. (**A**) Shows the structure of the network. (**B**) Shows the structure of the 1 × 50 or the 1 × 16 2-D convolutional layers in (**A**,**C**) shows the structure of the 1 × 3, 1 × 5, and 1 × 7 2-D convolution layers and 3, 5, and 7 1-D convolution layers in (**A**) Cov, convolution; Concat, concatenate; ReLU, rectified linear unit; BatchNorm: batch normalization.

The basic algorithm for this research, which achieved binary classification between the ASD group and the normal group, could be summarized as a CNN with residual networks, as shown in Figure 4.

In Figure 4A, after preprocessed and transformed, the collected ECG data was input the model with the shape of 1 × 8 × 4,000, where 1 represented the number of channel, 8 corresponded to the height, and 4,000 corresponded to the width. After inputting it into the model, the model captured features from multi-lead signals using a 2-D convolution layer with a 1 × 50 convolution kernel initially. And then this layer output a 3-D tensor with the shape of 32 × 8 × 1,976, where 32 represented the number of channels, 8 corresponded to the height, and 1,976 corresponded to the width. This was followed by a 2-D convolutional layer with a 1 × 16 convolution kernel. And then this layer output a 3-D tensor with the shape of 32 × 8 × 242, where 32 represented the number of channels, 8 corresponded to the height, and 242 corresponded to the width. Subsequently, parallel 2-D convolutional layers with different kernel sizes (1 × 3, 1 × 5, and 1 × 7, representing different scales) were employed to extract multi-scale features from various leads. It was noticed that there were 6 1 × 3 2-D convolution layers, 5 1 × 5 2-D convolution layers, and 4 1 × 7 2-D convolution layers in this stage. And the shape of all of these convolution layers was 32 × 8 × 242 due to the different paddings. Each parallel feature obtained from the convolution layers was flattened into a 1-dimensional feature, and a 1-D block, utilizing the same kernel size, was applied to further extract features. During the process of being flattened, the corresponding parameters was 256 × 242, where 256 corresponded to the height, and 242 corresponded to the width. It was noticed that there were 4 3 1-D convolution layers, 4 5 1-D convolution layers, and 4 7 1-D convolution layers in this stage. An average pooling layer was then utilized after the 1-D block. Subsequently, we concatenated all the features extracted from the different parallel blocks and employed a fully connected layer as the classifier to yield the final outcome.

Reports indicated that in the early phases of signal extraction, the use of larger convolution kernels was advantageous since they had the capacity to encompass multiple facets of the signal in a single cycle (13). As the feature length decreased, the feasibility of employing smaller convolution kernels to capture finer details increased. Therefore, we implemented feature extraction by combining 3 different sizes of 2-D convolution layers and corresponding sizes of 1-D convolution layers in this model.

In Figure 4B, there were 2 kinds of convolution layers applied in this model. The upper layer was the convolutional layer applied to the 1 × 50 2-D convolution layer and 1 × 16 2-D convolution layer. In this block, from front to back, they were: batch normalization, rectified linear unit, max-pooling, convolution, max-pooling. Batch normalization was a technique that normalized the input to a neural network layer during training to improve convergence and reduce overfitting (14). Rectified linear unit was an activation function that introduced non-linearity in a neural network by setting negative values to zero, allowing the network to learn complex patterns (15). Max-pooling was a down-sampling operation that extracted the most significant information from a region of the input by selecting the maximum value, reducing the spatial dimensions of the data (16). Convolution was an operation that extracted features from input data by applying a set of learnable filters to create feature maps, which were essential for image and pattern recognition tasks (17). The lower layer was the convolutional layer applied to the 1 × 3, 1 × 5, and 1 × 7 2-D convolution layers and 3, 5, and 7 1-D convolution layers. In this block, from front to back, they were: convolution, batch normalization, rectified linear unit, convolution, batch normalization, convolution, batch normalization, dropout. Dropout was a neural network regularization method that randomly deactivated a portion of neurons during training to enhance model generalization and prevent overfitting (18). From the input end to the output end, there was a short connection, which was a residual network.

Our framework was implemented based on PyTorch and ran on the NVIDIA Corporation GV100GL (Tesla V100 SXM2 32GB) graphics card. An effective optimization method named Adam was adopted to achieve efficient computing (19). A weighted loss function named cross entropy was adopted during the training to achieve better performance by focusing on the samples that were not easily classified (20). The hyperparameters of our proposed CNN were set to {53, 0.001, 500}*,* which denoted the batch size, learning rate, and training epoch respectively. More details were in the Supplementary Excel S1.

### 2.3. Statistical method

We utilized non-parametric tests including the Chi-square test and Fisher's precision test to assess the association between qualitative data presented in our tables. Additionally, we analysed quantitative data, such as age, using the Mann–Whitney *U* test. SPSS Statistics 26 was used as the statistical software, with significance defined as *P* < 0.05. To evaluate the performance of our AI model, we assessed the prediction accuracy, specificity, sensitivity, precision, area under the receiver operating characteristic curve (AUC), and F1 score (harmonic mean of the predictive positive value and sensitivity). Confusion matrices were utilized to illustrate the details of the calculation process, with prediction accuracy, sensitivity, specificity, and F1 score for each class determined by accuracy, true-positive (TP), true-negative (TN), false-positive (FP), and false-negative rates (FN), using the following formulas. Further information regarding these methods is available in our previous study (21):

(1) $$\mathrm{Accuracy}=\frac{\mathrm{TP}+\mathrm{TN}}{\mathrm{TP}+\mathrm{TN}+\mathrm{FN}+\mathrm{TP}}$$

(2) $$\mathrm{Precision}=\frac{\mathrm{TP}}{\mathrm{TP}+\mathrm{FP}}$$

(3) $$\mathrm{Sensitivity}(\mathrm{Recall})=\frac{\mathrm{TP}}{\mathrm{TP}+\mathrm{FN}}$$

(4) $$\mathrm{Specificity}=\frac{\mathrm{TN}}{\mathrm{FP}+\mathrm{TN}}$$

(5) $$F1\;\mathrm{score}=\frac{2\times \mathrm{sensitivity}\times \mathrm{precision}}{\mathrm{sensitivity}+\mathrm{precision}}$$

## 3. Results

We summarized the basic characteristics of the ASD patients in the training–validation dataset in Table 2. Females accounted for 76.6% of patients, average age was 41.9 ± 13.7 years old. In the normal heart group, females accounted for 55.2% of patients. The average age was 47.3 ± 17.7 years old. The secundum ASD group consisted of 387 individuals, accounting for 98.7% of the total. Primum ASD was more likely to be associated with mitral and tricuspid valve insufficiency than secundum ASD. Meanwhile, in the external test dataset, female participants accounted for 74% of the ASD group. The average age was 54.8 ± 16.3 years old. The secundum ASD group consisted of 49 individuals, accounting for 98% of the total, while in the normal heart group, females accounted for 67.3%. The average age was 44.3 ± 13.1 years old.

**Table 2:** Characteristics of the sample, ASD group in the training-validation group.

	Total (*N* = 401)	Secundum ASD (*n* = 387)	Primum ASD (*n* = 14)	*P* value
Age, mean (SD)	41.9 ± 13.7	41.8 ± 13.7	43.3 ± 13.8	0.701
Female, *n* (%)	307 (76.6)	298 (77.0)	9 (64.3)	0.099
Tricuspid incompetence, *n* (%)	46 (11.5)	37 (9.6)	9 (64.3)	**<0**.**001**
Hypertension, *n* (%)	29 (7.2)	28 (7.2)	1 (7.1)	0.99
Mitral incompetence, *n* (%)	25 (6.2)	17 (4.4)	8 (57.1)	**<0**.**001**
Cerebrovascular disease, *n* (%)	12 (3.0)	12 (3.1)	0	0.649
Coronary heart disease, *n* (%)	11 (2.7)	10 (2.6)	1 (7.1)	0.327
NYHY III–IV, *n* (%)	9 (2.2)	9 (2.3)	0	0.724
Diabetes, *n* (%)	7 (1.7)	6 (1.6)	1 (7.1)	0.222
Hyperthyroidism, *n* (%)	3 (0.7)	3 (0.8)	0	0.899
Aortic incompetence, *n* (%)	1 (0.2)	1 (0.3)	0	0.965
Dilated cardiomyopathy, *n* (%)	1 (0.2)	1 (0.3)	0	0.965
Hypertrophic cardiomyopathy, *n* (%)	1 (0.2)	1 (0.3)	0	0.965

ASD, atrial septal defect; NYHY III–IV, New York Heart Association classification of heart failure III–IV.

The bold values indicate statistically significant difference between the two groups.

**Figure 5:**
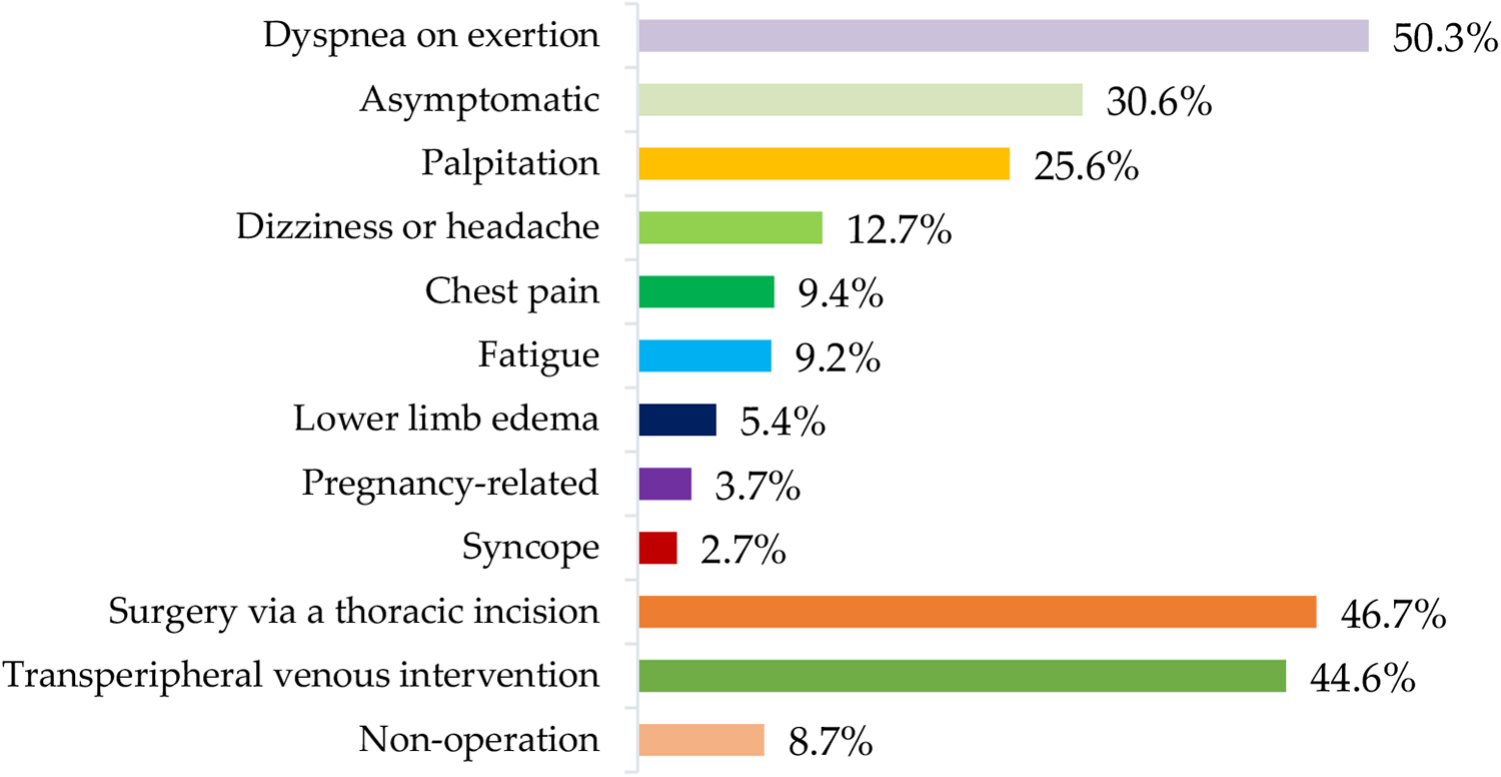
Distribution of symptoms and treatments of the ASD group in the training-validation dataset. ASD, atrial septal defect.

Out of 401 cases in the training-validation dataset, 202 (50.3%) experienced dyspnea on exertion, 123 (30.6%) were asymptomatic, 103 (25.6%) had palpitation, 51 (12.7%) had dizziness or headache, 38 (9.4%) experienced chest pain, 37 (9.2%) reported fatigue, 22 (5.4%) had lower limb edema, and 15 (3.7%) were pregnancy-related. Additionally, 11 (2.7%) experienced syncope. Among these cases, 187 (46.7%) underwent surgery via a thoracic incision, 179 (44.6%) received trans-peripheral venous intervention, and 35 (8.7%) did not undergo any surgery. Figure 5 provided a systematic description of symptoms and treatments in the ASD group in the training–validation dataset.

Meanwhile, we also summarized the ECG characteristics of the ASD group and normal group, which were shown in Table 1. In the training-validation dataset, we found significant differences between the two groups in terms of sinus rhythm(*p* < 0.001), atrial rhythm (*p* < 0.001), first-degree atrioventricular block (1°AVB) (*p* < 0.001), incomplete right bundle branch block (IRBBB) (*p* < 0.001), CRBBB (complete right bundle branch block) (*p* < 0.001), enlargement of the left atrium (ELLA) (*p* < 0.001), enlargement of the right atrium (ELRA) (*p* < 0.001), and RVH (right ventricular hypertrophy) (*p* < 0.001), atrial premature beat (APB) (*p* < 0.05) and ventricular premature beat (VPB) (*p* < 0.001). In the external test dataset, we found significant differences in sinus rhythm (*p* < 0.05), atrial rhythm (*p* < 0.05), CRBBB (*p* < 0.05), IRBBB (*p* < 0.05), and VPB (*p* < 0.05).

Table 3 showed the AI performance for the ASD group and normal group. In the training–validation dataset, the accuracy of identification for the AI-ECGs of the ASD group was 0.97, with a precision of 0.90, recall of 0.97, specificity of 0.97, F1 of 0.93, and AUC of 0.99. Meanwhile, in the test dataset, the accuracy of identification for AI-ECG of the ASD group was 0.82, with a precision of 0.90, recall of 0.74, specificity of 0.91, F1 of 0.81, and AUC of 0.87.

**Table 3:** Demonstration of the AI-ECG performance in the ASD group and normal group.

	Training-validation dataset		Test dataset	
	ASD group (*n* = 526)	Normal group (*n* = 2,124)	ASD group (*n* = 50)	Normal group (*n* = 46)
Accuracy	0.97	0.97	0.82	0.82
Precision	0.90	0.99	0.90	0.76
Sensitivity/recall	0.97	0.97	0.74	0.91
Specificity	0.97	0.97	0.91	0.74
F1	0.93	0.98	0.81	0.83
AUC	0.99	0.99	0.87	0.87

ASD, atrial septal defect; AI, artificial intelligence; AUC, area under curve.

**Figure 6:**
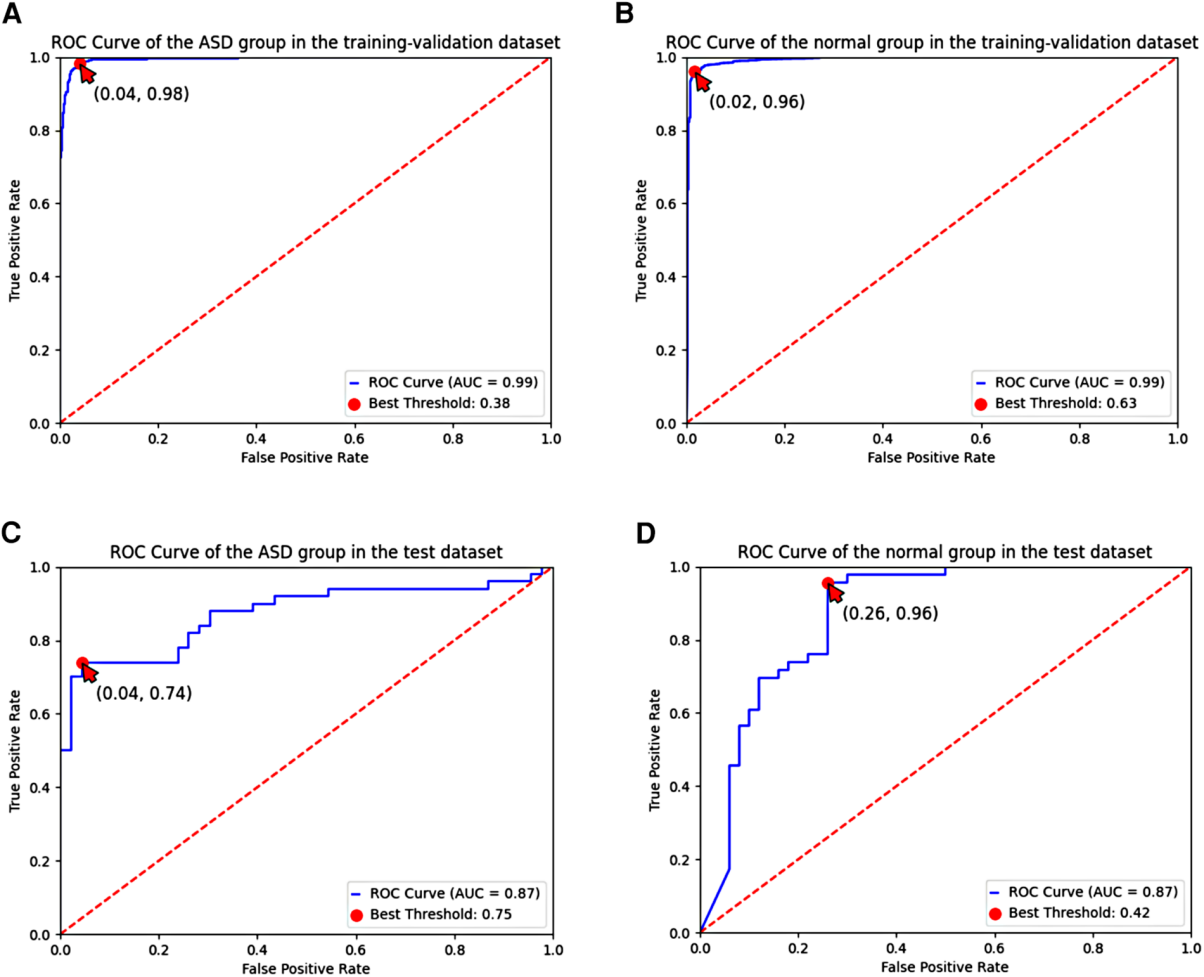
ROC curve of the ASD group and the normal group based on the 8-lead ECG; (**A**,**B**) demonstrated the ROC curve of the classification of the ASD group and the normal group in the training-validation dataset; (**C**,**D)** demonstrated the ROC curve of the classification of the ASD group and the normal group in the test dataset. ROC, receiver operating characteristic; ASD, atrial septal defect.

**Figure 7:**
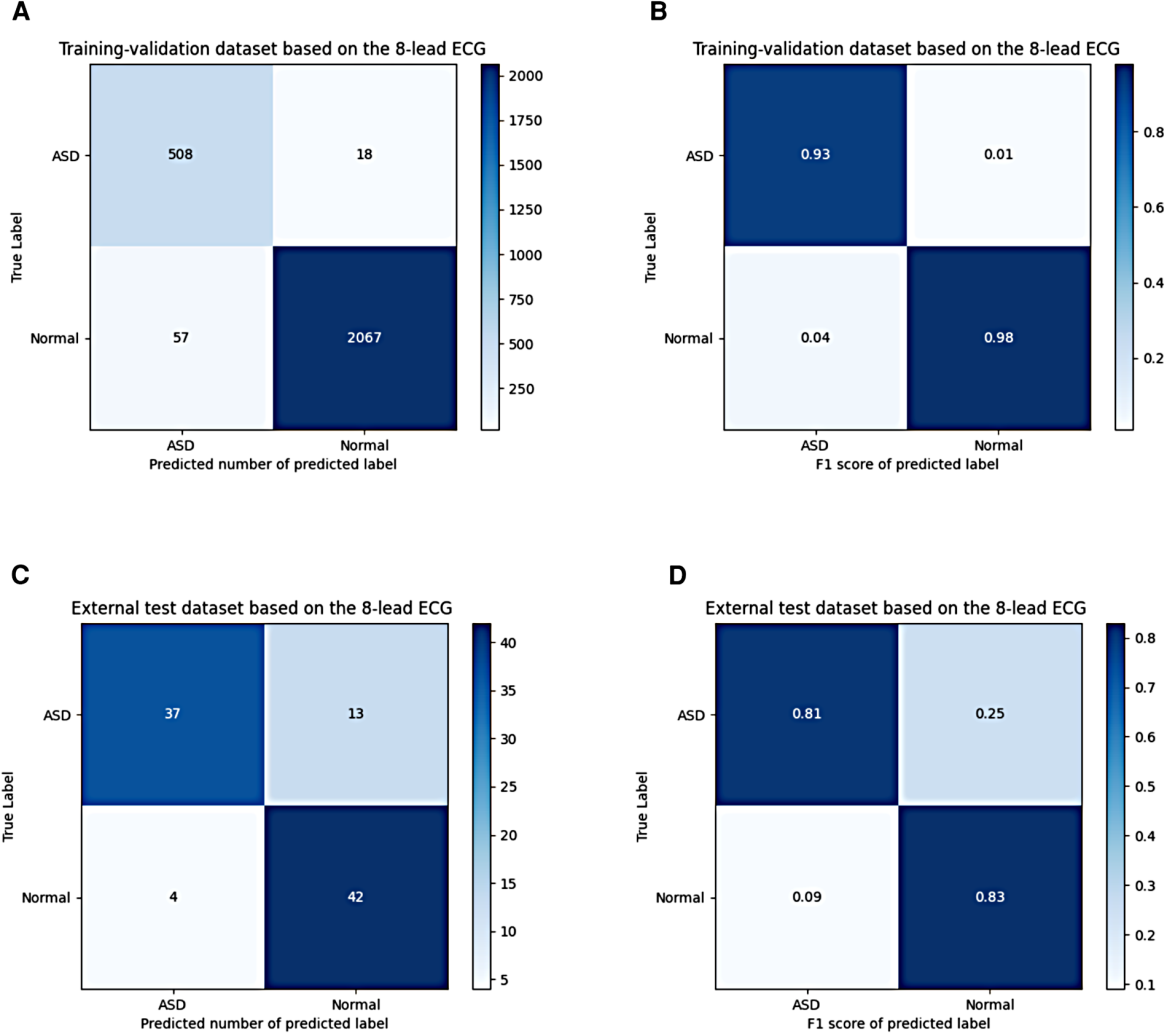
Confusion matrix of the ASD group and the normal group based on the 8-lead ECG; (**A**,**B**) shows the confusion matrix of the predicted number and the F1 score in the training-validation dataset; **(C**,**D**) shows the confusion matrix of the predicted number and the F1 score in the test dataset. ASD, atrial septal defect.

These results were shown in receiver operating characteristic (ROC) curves and confusion matrices, which could be found in Figures 6, 7, providing details of the AI model based on 8-lead ECG. Figure 6A demonstrated the ROC curve of the classification for the ASD group in the training-validation dataset with the AUC of 0.99. And the AUC of the normal group was 0.99 shown in the Figure 6B. Meanwhile, Figure 6C demonstrated the ROC curve of the classification of the ASD group in the external test dataset with the AUC of 0.87. And the AUC of the normal group was 0.87 shown in the Figure 6D. Figure 7 showed the confusion matrix of the ASD group and normal group. Figures 7A,C showed the overall predicted results of the model with the 10-fold cross-validation. Figures 7B,D showed the F1 scores of corresponding parts in Figures 7A,C which were consistent with Table 3.

Table 4 presented a comparative analysis of AI performance between subgroups of the ASD group and the normal group. In the subgroup analysis represented in Table 4, the classification of two subgroups with the electrocardiographic features of IRBBB and CRBBB, where the F1 score was 0.98, demonstrated the best F1 score. The matrices were shown in the Supplementary Figure S1, which supported our results.

**Table 4:** AI -ECG performance comparison between subgroups of the ASD group and the normal group.

	Normal (n:121, 1,514)	RVH (n:22, 9)	IRBBB (n:191, 53)	CRBBB (n:72, 30)	Training-validation dataset (n:526, 2,124)
Accuracy	0.97	0.94	0.97	0.97	0.97
Precision	0.70	0.95	0.98	0.96	0.90
Sensitivity/recall	0.92	0.95	0.98	1.00	0.97
F1	0.80	0.95	0.98	0.98	0.93
AUC	0.99	0.99	0.99	0.99	0.99

The number of electrocardiograms indicating atrial septal defect is represented before the comma in parentheses, and the number of electrocardiograms in the normal group is represented after the comma. ASD, atrial septal defect; AI, artificial intelligence; AUC, area under curve; IRBBB, incomplete right bundle branch block; CRBBB, complete right bundle branch block; RVH, right ventricular hypertrophy.

## 4. Discussion

In this study, we presented an AI model for detecting ASD based on ECG data. Our study involved a total of 3,005 ECGs and 2,880 participants aged 18 and older, collected from two medical centres. Specifically, Wuhan Tongji Hospital contributed 2,903 ECGs and 2,778 participants. After excluding 253 ECGs and 253 participants from Tongji Hospital, our dataset consisted of 526 ECGs from 401 individuals with ASD and 2,124 ECGs from a control group of 2,124 individuals with normal cardiac structures. Our method demonstrated a strong discriminatory ability in distinguishing between ASD cases and those with normal heart structures, achieving an AUC of 0.99 and an F1 score of 0.93. In an external test dataset, which included 50 ECGs from 50 participants with ASD and 46 ECGs from 46 participants with normal heart structures, our model achieved an AUC of 0.87 for distinguishing ASD cases and an F1 score of 0.81.

Comparatively, a previous study by Moris et al. (9) reported an AUC of 0.96, assessing 1,192 ECGs (828 from individuals with normally structured hearts and 364 with ASD) involving 792 participants under the age of 18. Another study by Liu et al. (10) achieved an AUC of 0.88, identifying ASD in 1,196 patients of all age ranges with secundum ASD, along with a control group of 21,430 individuals, using a CNN-based model. In contrast, our study, conducted by Miura et al. (11), successfully identified ASD across three medical institutions on two continents, using 671,201 ECGs from 80,947 patients aged 18 and older, incorporating a CNN-based model with an AUC ranging from 0.85 to 0.90.Moris et al. (9) focused on image classification in the pediatric population. Additionally, while Liu et al. (10) focused exclusively on secundum ASD, our study also included premium ASD other than secundum ASD in the model development process. Miura et al (11). definitely did excellent work which involving 3 centres from 2 continents. However, all these studies utilized 12-lead ECG data. Therefore, the novelty of our study lay in the utilization of artificial intelligence-enabled 8-lead ECG signal classification, which offered a lower computational load compared to 12-lead ECGs, for ASD screening in adults. While it was worth noting that AI-ECG models for ASD detection had been developed by researchers worldwide in recent years, only the model developed by Miura et al. (11). and our model involved multiple medical centres to validate their robustness.

Table 2 demonstrated that premium ASD patients had a significantly higher likelihood of associated with mitral and tricuspid insufficiency compared to secundum ASD patients (*p* < 0.05), which might be due to the difference in the anatomical characteristics of the two types of ASD (1). This physiological change provided hope and potential for the classification of subtypes of ASD based on the AI-ECG. Our study displayed the basic data and electrocardiogram data of the experimental group's two types of ASD (Supplementary Table S1). Nonetheless, as the majority of cases involved secundum ASD, their higher incidence limits the scope of development for the AI-ECG model for the subgroups of ASD. If there are larger sample sizes for the two types of ASD, more meaningful classification criteria, and the availability of multimodal data, it may also be possible to achieve classification of the subtypes of ASD.

Detecting and treating ASD at an early stage is significantly important for delaying the associated heart failure and pulmonary hypertension, preventing malignant arrhythmias and related complications, and ultimately improving patient prognosis (1, 2). Figure 5 showed the distribution of symptoms and treatments of the ASD group in the training-validation dataset. Although the description of symptoms was influenced by subjective factors, these symptoms were often associated with the severity of the atrial defect. Therefore, the statistical results of this study served as a reminder of the severity of the ASD group in this training–validation set. Meanwhile, the gold standard for diagnosing ASD—echocardiography—is difficult to implement as a screening tool in some regions and institutions due to financial reasons. In comparison, ECG has the advantages of being non-invasive and inexpensive. Studies have shown that patients with ASD display ECG changes, such as 1°AVB, RBBB, and RVH, when compared to the normal population (4). However, ASD cannot be solely diagnosed based on these electrocardiographic features. Our study utilized the network to extract ECG features from the training set and then make predictions for the remaining ECGs. The results showed that AI-ECG had excellent performance in classifying the ASD group and the normal group, providing strong theoretical support for applying AI-ECG in ASD screening. Moreover, congenital heart disease is a condition that can increase circulatory load and is a risk factor for high-risk pregnancies. Therefore, detection of ASD in adults has significant implications for successful pregnancy and delivery. Hence, the related results could serve as an efficient reference for clinical decision-making (4). With the application of relevant research worldwide, using AI-ECG as an automated screening tool for ASD, whether in routine examinations or wearable intelligent devices, is bound to bring significant economic and social benefits.

When processing ECG data, imbalanced samples are often encountered where the number of normal samples is much higher than that of abnormal samples. This issue was observed in the AI model for hypertrophic cardiomyopathy in an earlier study by Ko et al. (22). In our own study, the ratio of normal to abnormal samples was approximately 4:1, with 2,124 normal samples and 526 abnormal samples. Given that the incidence of ASD is 1.7 per 1,000 births (23), this imbalance is understandable. However, it can lead to the model over-focusing on normal samples. To mitigate the issue of overfitting, we implemented dropout, batch normalization, and cross-validation techniques. As for the data augmentation method, we once experimented with the cross-sensitive approach, and the formula was included in the code. However, we observed that the results were similar to those of the previous experiment. To maintain the simplicity of the model structure, we ultimately decided not to use it. Meanwhile as shown in Table 1. We found significant differences between the two groups in terms of atrial rhythm (*p* < 0.05), 1°AVB (*p* < 0.05), IRBBB (*p* < 0.05), CRBBB (*p* < 0.05), ELLA (*p* < 0.05), ELRA (*p* < 0.05), and RVH (*p* < 0.05). All of these findings match the characteristics of previous studies on ECG manifestations of ASD (24–27) which could be an interpretation for doctors to understand the design of this study. Furthermore, despite the sample imbalance, our study yielded favourable classification performance both within our hospital and at external medical institutions. Therefore, our study is still feasible and valuable.

As you can see, we conducted subgroup analyses in Table 4. The purpose of designing this experiment shown in Table 4 can be summarized in two main objectives: Firstly, in real-world medical practice, we often encounter scenarios where we need to distinguish ASD from other conditions such as right ventricular hypertrophy or complete right bundle branch block, as they may manifest similar electrocardiographic patterns. This experiment was designed to assess the model's capability to accurately differentiate between ASD and these related conditions. Secondly, especially in the context of large-scale health screenings, a substantial number of normal electrocardiograms are typically present within the screened population. Our study included sizable samples of normal electrocardiograms in both the training-validation and test datasets. Therefore, discussing this scenario serves as a necessary supplementary aspect of our research. While discussions involving more medical centres and larger sample sizes would certainly provide valuable insights, the subgroup analysis presented in Table 4 effectively shows the model's classification performance in the aforementioned scenarios. And the confusion matrix related to Table 4 was shown in the Supplementary Figure S1.

Our AI model was based on ECG signals. It is known that the ECG could be presented in two different forms: images and signals. The characteristics of images were more intuitive. The characteristics of signals were more convenient to transmit and process. Therefore, AI-ECG based on signals had indispensable advantages compared with the model based on images. Moreover, to improve the efficiency of the calculation, the raw data we fed to the model was from 8-lead ECGs rather than the traditional 12-lead ECGs. A previous study encouraged these attempts (28). Our study showed the application potential of AI-enabled 8-lead ECG in this field besides traditional arrhythmia. In addition, the results of our study shown in the Table 5, namely the F1 values and other scores, proved the feasibility of our attempts. And the related confusion matrix was shown in the Supplementary Figure S2. Meanwhile, although the traditional CNN without residual networks has already been a relatively mature AI architecture, the previous study also proved the advantages of CNN with residual networks (8). Therefore, we chose the CNN with residual networks to increase the gradients of the network to improve the performance.

**Table 5:** AI performance of different ECG leads in the external test.

	12 leads		8 leads	
	ASD group (*n* = 50)	Normal group (*n* = 46)	ASD group (*n* = 50)	Normal group (*n* = 46)
Accuracy	0.82	0.82	0.82	0.82
Precision	0.86	0.77	0.90	0.76
Sensitivity/recall	0.76	0.87	0.74	0.91
Specificity	0.87	0.76	0.91	0.74
F1	0.81	0.82	0.81	0.83
AUC	0.87	0.87	0.87	0.87

ASD, atrial septal defect; AI, artificial intelligence; AUC, area under curve; ECG, electrocardiogram.

### 4.1. Limitation

While this study involved data from two centres, having more centres and a larger dataset could further enhance the robustness of the model. Additionally, there was a lack of supporting interpretative elements such as heatmaps. Furthermore, there was a lack of human-machine performance comparison.

## 5. Conclusions

An ECG-based detection of ASD using an artificial intelligence algorithm can be achieved with high diagnostic performance, and it shows great clinical promise. Our research on AI-enabled 8-lead ECG detection of ASD in adults is expected to provide robust references for early detection of ASD, healthy pregnancies, and related decision-making. A lower number of leads is also more favourable for the application of portable devices. As future research delves deeper into this technology and discussions regarding its applicability in various contexts expand, it is expected that this technology will bring significant economic and societal benefits.

## Data Availability

The raw data supporting the conclusions of this article will be made available by the authors, without undue reservation.
