# Supplementary material for: Artificial intelligence-enabled 8-lead ECG detection of atrial septal defect among adults: a novel diagnostic tool
Source: Front Cardiovasc Med. 2023 Nov 13;10:1279324. doi: 10.3389/fcvm.2023.1279324 (PMC10679442; doi:10.3389/fcvm.2023.1279324)
Supplement: Supplementary file 2 [file Datasheet1.pdf]

Figures

A

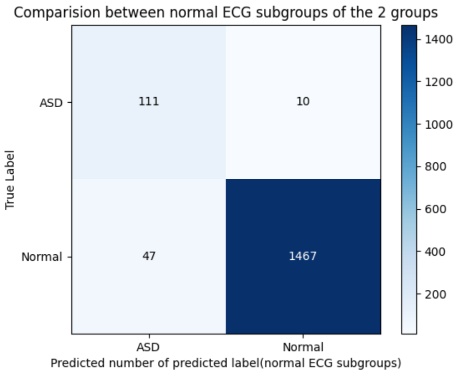

B

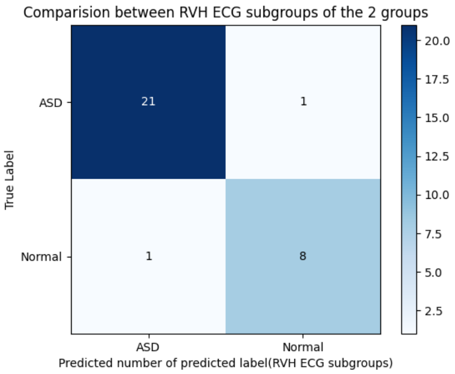

C

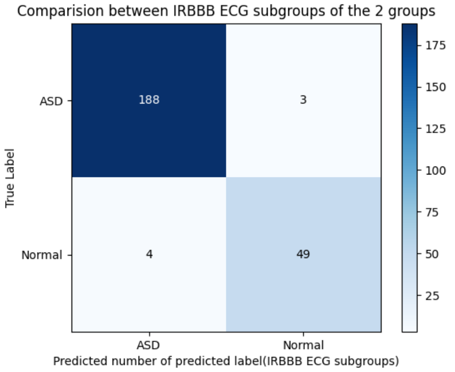

D

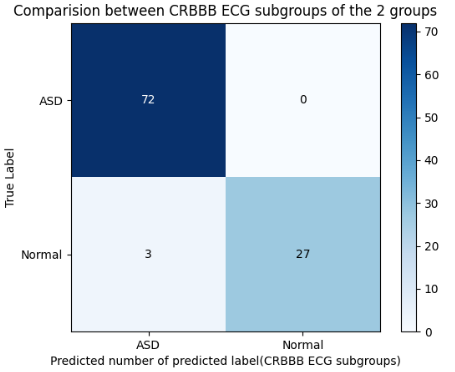

Figure S1

Confusion matrix for Table 4;**(A)** shows the confusion matrix of the predicted number in the comparison between the normal ECG subgroups of the ASD group and the normal group. (**B)** shows the confusion matrix of the predicted number in the comparison between the RVH ECG subgroups of the ASD group and the normal group;(**C)** shows the confusion matrix of the predicted number in the comparison between the IRBBB ECG subgroups of the ASD group and the normal group. (**D)** shows the confusion matrix of the predicted number in the comparison between the CRBBB ECG subgroups of the ASD group and the normal group. ASD, atrial septal defect; ECG, electrocardiogram; IRBBB, incomplete right bundle branch block; CRBBB: complete right bundle branch block; RVH, right ventricular hypertrophy; ASD, atrial septal defect; ECG, electrocardiogram; IRBBB, incomplete right bundle branch block; CRBBB, complete right bundle branch block; RVH, right ventricular hypertrophy;

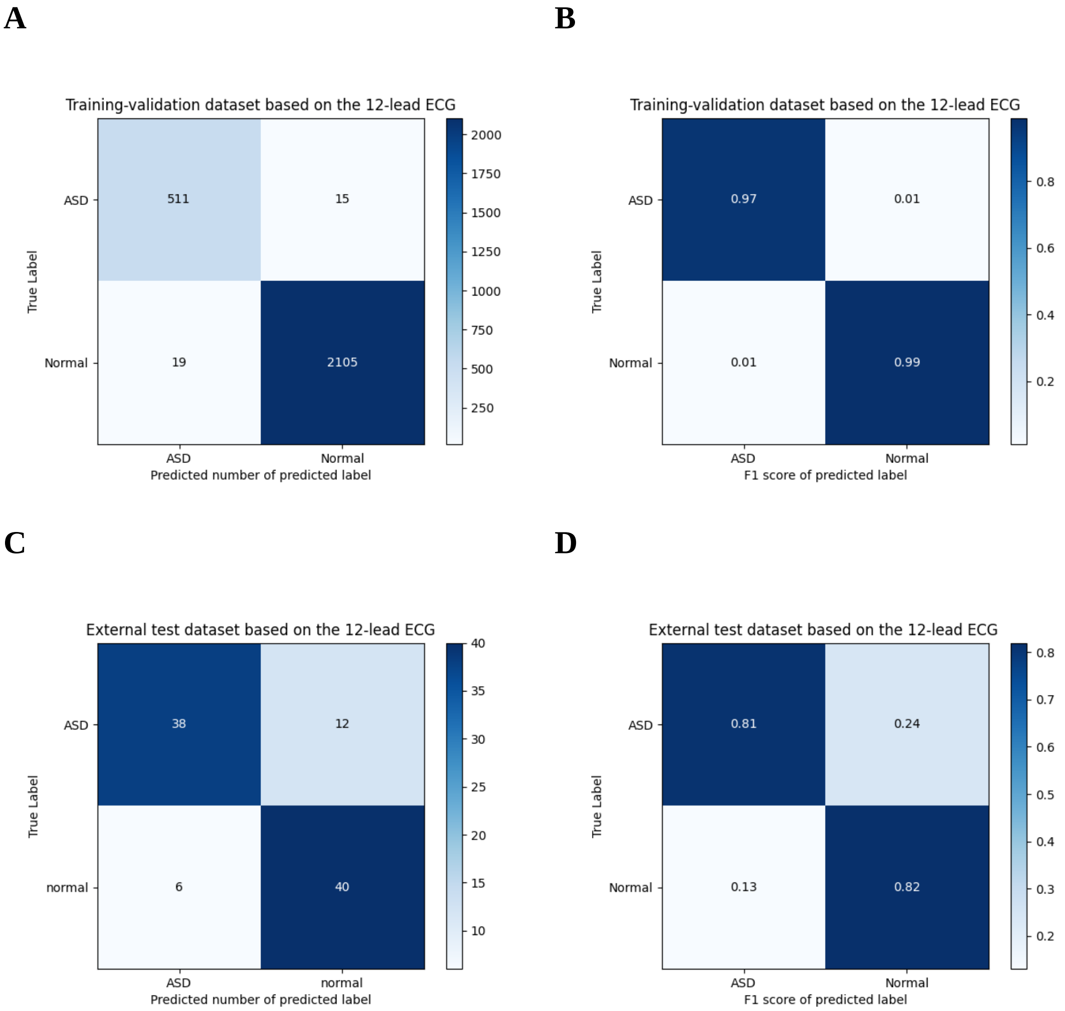

Figure S2

Confusion matrix of the ASD group and the normal group based on the 12-lead ECG;**(A, B)** shows the confusion matrix of the predicted number and the F1 score in the training-validation dataset;**(C, D)** shows the confusion matrix of the predicted number and the F1 score in the test dataset. ASD, atrial septal defect; ECG: electrocardiogram.

This is a provisional file, not the final typeset article

Tables

Table S1 ECG features of the subgroups in the training-validation dataset.

	Secundum atrial septal defect (n=387)	Primum atrial septal defect (n=17)	P value
Total	510(97.0)	16(3.0)	-
Normal ECGs	120(23.5)	1(6.3)	0.085
Abnormal ECGs	390(76.5)	15(93.8)	0.085
Sinus rhythm	450(88.2)	12(75.0)	0.118
Atrial rhythm	60(11.8)	4(25.0)	0.118
1°AVB	28(5.5)	2(12.5)	0.230
IRBBB	217(45.5)	7(43.8)	0.924
CRBBB	81(15.9)	5(31.3)	0.063
RVH	66(3.1)	1(6.3)	0.373
LVH	8(1.6)	0	0.780
ELLA	17(3.3)	1(6.3)	0.412
ELRA	10(2.0)	0	0.732
APB	17(3.3)	0	0.587
VPB	22(4.3)	2(12.5)	0.162

Notes: ASD, atrial septal defect; ECG, electrocardiogram; Atrial rhythm, atrial fibrillation, atrial flutter, and atrial tachycardia; 1°AVB, first-degree atrioventricular block; IRBBB, incomplete right bundle branch block; CRBBB, complete right bundle branch block; RVH, right ventricular hypertrophy; LVH, left ventricular hypertrophy; ELLA, enlargement of the left atrium; ELRA, enlargement of the right atrium; APB, atrial premature beat; VPB, ventricular premature beat.
